# Optimization of Radio-Frequency Heating Conditions to Achieve Uniform Pasteurization of Irregularly Shaped Spices: The Case of Star Anise

**DOI:** 10.3390/foods15152621

**Published:** 2026-07-27

**Authors:** Yuxuan Zhang, Xingfang Mao, Dingting Zhou, Yingqi Tian, Teng Cheng, Xiaoming Lu, Xiangyu Guan

**Affiliations:** 1 Key Laboratory of Food Nutrition and Healthy in Universities of Shandong, College of Food Science and Engineering, Shandong Agricultural University, Tai’an 271018, China; 2025121112@sdau.edu.cn (Y.Z.); 2024121022@sdau.edu.cn (X.M.); 2 College of Agronomy and Life Sciences, Zhaotong University, Zhaotong 657000, China; 20260005@ztu.edu.cn; 3 College of Mechanical and Electronic Engineering, Northwest A&F University, Yangling 712100, China; tianyingqi@nwafu.edu.cn; 4 College of Food and Bioengineering, Zhengzhou University of Light Industry, Zhengzhou 450001, China; tengcheng@zzuli.edu.cn; 5 Key Laboratory of Cold Chain Food Processing and Safety Control, Ministry of Education, Zhengzhou University of Light Industry, Zhengzhou 450001, China

**Keywords:** packaging material, arrangement of star anise, packaging bag placement, RF heating uniformity, process optimization

## Abstract

Radio-frequency (RF) heating has been proven effective in inactivating foodborne pathogens or fungi in spices. However, non-uniformity heating remains a major obstacle limiting RF treatment application in the commercial pasteurization of irregularly shaped spices. This study evaluated the effects of star anise arrangement, packaging materials, and dual-bag placement on RF heating uniformity after identifying the optimal input power and electrode gap. The optimal conditions were 400 W and an electrode gap of 5 cm. Under these conditions, heating uniformity was significantly improved by a staggered arrangement, using bags with 0.16 mm aluminum foil (plastic/foil composite) and face-to-face placement of two bags; the average heating uniformity index of the stacked packages decreased from 0.30 to 0.135, corresponding to a reduction of approximately 55.0%. These findings provide practical guidance for scalable, eco-friendly RF pasteurization of irregular spice products, emphasizing key considerations in sample arrangement and packaging/bag placement to control heating uniformity.

## 1. Introduction

China is a major producer, consumer, and exporter of spices worldwide. Star anise (*Illicium verum* Hook. *f.*) is a representative irregularly shaped spice with a distinctive star-shaped structure, typically consisting of eight capsules with variable geometry. As a traditional and important spice, star anise is widely used as a food seasoning owing to its characteristic aroma. [1]. Although spices are generally characterized by low moisture content and are often considered unfavorable for microbial growth, microbial contamination may occur throughout the production chain, including cultivation, harvesting, processing, transportation, and storage [2,3]. Therefore, effective control of microorganisms, particularly mold contamination such as Aspergillus spp., is important for ensuring the safety and quality of star anise.

Traditional sterilization methods rely on high temperatures in either dry or moist media to denature microbial proteins, causing their death. However, high temperatures or secondary drying after humid heating significantly degrades thermolabile bioactive compounds in star anise, reducing nutritional quality. Conventional pasteurization alternatives (chemical, ozone, UV-C, irradiation) fail to meet star anise’s stringent requirements due to fundamental limitations: chemical residue, incomplete penetration in irregular geometries, and consumer concerns. Radio-frequency (RF) heating is a non-ionizing electromagnetic method capable of volumetric heating through dipole rotation/ion polarization, offering rapid and controllable processing with deep penetration [4]. Studies have now reported that RF heating can significantly reduce the number of foodborne pathogens in spices. Wei et al. found that RF treatment reduced *Salmonella* levels in whole black peppercorns and ground black pepper by 5.31 and 5.98 log CFU/g, respectively [5]. Kim et al. used 27.12 MHz RF heating to treat black pepper and red chili peppers [6]. The results showed that after 50 s of RF treatment, *Salmonella Typhimurium* and *Escherichia coli* O157 in black pepper were reduced by 2.80–4.29 log CFU/g; after 40 s of RF treatment, pathogenic bacteria in red chili peppers were reduced by 3.38 to >5 log CFU/g [7]. Recent studies indicated that RF heating was a feasible alternative to octagonal pasteurization, capable of preserving the nutritional value of spices [8,9,10].

Although RF heating has been applied in pasteurization of spices, its technical limitation is the uneven temperature distribution within food materials. Heating non-uniformity is commonly associated with differences in dielectric properties between the product and surrounding medium, edge effects, sample geometry, moisture distribution, product arrangement, packaging materials, and RF operating parameters such as power level and electrode gap [11,12,13,14,15]. In particular, regions with sharp edges or relatively high moisture content may absorb RF energy more readily, resulting in localized temperature increases, whereas other regions may heat more slowly. Such uneven heating can lead to insufficient microbial inactivation in colder regions or quality degradation in overheated areas, thereby limiting the practical application of RF pasteurization [16,17].

Star anise represents a particularly challenging material for RF pasteurization because of its distinctive eight-pointed morphology and heterogeneous structure. The pointed capsules and irregular spatial arrangement may promote localized field enhancement and uneven energy absorption, making it difficult to achieve uniform heating throughout the product. However, the existing literature on RF heating uniformity predominantly focuses on powdered spices or uniform geometric materials (grains, seeds), leaving a substantial research gap regarding irregularly shaped spices, particularly star anise [11,18]. Meanwhile, the effects of star anise arrangement pattern, packaging material, dual-package placement configuration, and RF operating conditions on heating rate and temperature uniformity remain insufficiently characterized. Moreover, RF heating at 70 °C (cold spot) was reported to achieve a 4-log population reduction in *Aspergillus flavus* with no significant quality loss, and drying studies showed higher volatile oil/trans-anethole retention at 70 °C than at 60/80 °C [19,20]. Therefore, 70 °C was selected as the optimal target temperature to balance microbial inactivation and preservation of thermolabile bioactive compounds.

The objectives of this study were to (1) determine suitable RF input power and electrode gap for heating star anise to a target surface temperature of 70 °C, providing a basis for potential RF pasteurization applications; (2) evaluate how star anise arrangement patterns affect heating uniformity; (3) assess the impact of common packaging materials on RF heating characteristics; and (4) optimize dual-packaging configurations for commercial-scale RF processing.

## 2. Materials and Methods

### 2.1. Materials

Star anise was purchased from a local market in Taishan District (Tai’an, Shandong Province, China). Samples were manually sorted for consistent size (maximum diagonal: 3.98 ± 0.36 cm; minimum diagonal: 3.37 ± 0.30 cm; height: 0.92 ± 0.09 cm) to ensure experimental reproducibility. Test materials were categorized into three morphological fractions: whole star anise (with seeds intact), de-seeded star anise (seeds removed), and isolated star anise seed, enabling investigation of moisture content variation across morphological components. Commercially available packaging materials commonly used for spice distribution were evaluated: kraft paper bags, double-sided plastic polyethylene bags (0.08 mm thickness), and composite bags with plastic on one side and 0.16 mm aluminum foil on the opposite side.

### 2.2. RF Heating System

**Figure 1:**
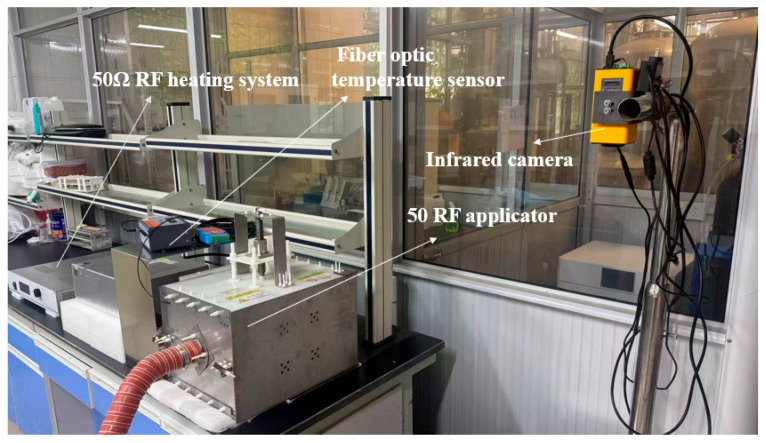
Actual diagram of the main instruments and equipment used in the experiment.

A newly self-built small-scale 50 Ω RF heating system (1–1000 W, 13.56 MHz) with electrode adjustment function was used for heat treatments (Figure 1). The RF generator, automatic matching box (MB), and 50 Ω RF applicator were connected through cables and a copper strip to form the RF system. The matching box detected changes in the sample between the electrodes, including variations in configuration, size, and temperature, and then its internal capacitances (C_L and C_T) were adjusted to restore the RF circuit output to an impedance-matching condition. The overall size of the small-scale RF system was similar to that of a current household microwave oven and the details of the RF system are described in Tian et al. [20].

### 2.3. Moisture Content and Dimension of Samples

Moisture content of de-seeded star anise, whole star anise, and isolated star anise seed was measured according to the China national standard (GB/T 7652-2016) [21] with modification. Three 3.2 ± 0.1 g replicate samples of each material type were placed in an aluminum dish and dried in a vacuum oven at 105 °C under pressure ≤ 0.1 kPa to a constant weight. Ten randomly selected star anise pods were measured using a transparent ruler to determine: (1) the longest axis (major diagonal), (2) the shortest axis (minor diagonal), and (3) the height (vertical dimension), with measurements recorded to 0.01 cm precision. Based on the preliminary experiments, star anise was arranged in a single layer within packaging bags, and the maximum weight per bag was determined to be 60 g at a uniform packing density.

### 2.4. Determining the Suitable RF Input Power and Electrode Gap

**Figure 2:**
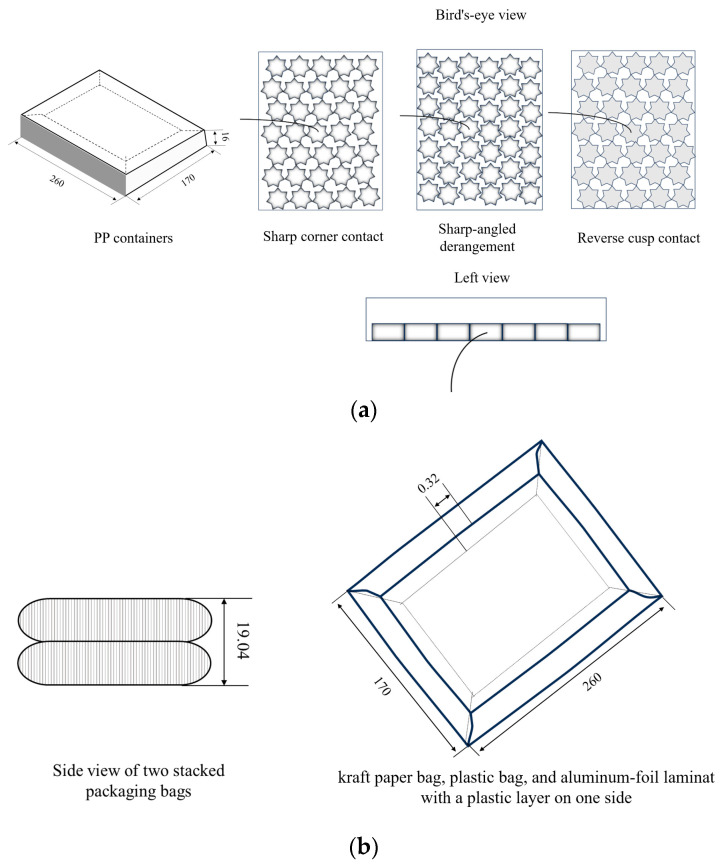
(**a**) Three stacking arrangement configurations and their multi-layer top/left/isometric views, and (**b**) side view of two stacked packaging bags; single package construction details showing the kraft paper bag, plastic inner lining, and aluminum foil laminated outer layer with dimensions. All dimensions are in mm.

To select the appropriate input power and electrode gap, a polypropylene container (26 cm long, 17 cm wide) with star anise (60 g) was placed on the center of the bottom electrode during the RF treatment (Figure 2a). Before the experiments, star anise samples were randomly selected and spread evenly in a single layer within a transparent container. Heating rates were conducted at RF input powers of 200, 400, and 600 W. At the appropriate RF input power, experiments were conducted to determine the most suitable electrode gap. The electrode gap increased from 3 to 7 cm at a 2 cm interval.

For each input power level, the target temperature of 70 °C was selected based on Zhang et al. (2021), who reported it as the optimal temperature for *A. flavus* inactivation during RF heating with no significant loss in quality [19]. Previous studies on star anise drying showed that, compared with 60 °C and 80 °C, treatment at 70 °C resulted in better retention of volatile oil and trans-anethole. Therefore, 70 °C was selected as the target temperature in this study to evaluate the RF heating behavior and temperature distribution uniformity of irregularly shaped star anise [22]. In addition, since the fixed heating time can be used to investigate the influence of adding the materials on RF heating uniformity [23], the RF heating time was set as 8 min or until reaching 70 °C (whichever occurred first). To attain the most suitable heating rate (4–6 °C/min) based on the temperature–time history among three RF input powers, the temperature of the star anise layer was measured at the geometric center using a fiber-optic temperature sensor (HQ-FTS-D120, Heqi Technologies Inc., Xian, China) with an accuracy of ±0.5 °C. The suitable input power and electrode gap were selected for further RF heating uniformity tests.

### 2.5. Evaluation of the Effect of Packaging Type on RF Heating Uniformity in Star Anise

#### 2.5.1. Effect of Different Star Anise Arrangements on RF Heating Uniformity

To systematically evaluate the influence of spatial arrangement in irregularly shaped spices on RF heating uniformity, star anise samples were configured into three distinct single-layer patterns within transparent plastic containers (26 cm × 17 cm): points touching, points staggered, and points touching but reversed (Figure 2a). The samples were not flipped, stirred, or rearranged during RF heating, and the initial arrangement was maintained throughout the treatment. Since the weight of star anise required to fill a consistent single layer varies significantly with these different spatial arrangements, 40, 75, and 40 g samples were utilized for each respective configuration based on the preliminary experiment. The initial surface temperature of each star anise arrangement was measured using an infrared camera (Fluke-RSE30, Fluke Corporation, Everett, WA, USA) with an accuracy of ±2 °C to establish a baseline temperature profile prior to RF treatment. After that, the prepared containers were placed in the center of the bottom electrode and heated under the suitable RF input power (400 W) and electrode gap (5 cm). Samples were treated for 8 min or until reaching 70 °C (whichever occurred first) in the RF cavity. The thermal imaging measurements for the different spatial arrangements of star anise were mapped successively and immediately after the RF treatment was completed and they were finished within 30 s to reduce temperature loss according to the methods reported by Guan with modification [24].

#### 2.5.2. Effect of Different Packaging Materials on RF Heating Uniformity

To determine the effect of the packaging materials for star anise on the RF heating rate and uniformity, three types of packaging materials (kraft paper, double-sided plastic, and one-sided plastic/one-sided aluminum foil bags, 26 cm × 17 cm × 0.16 mm) were selected (Figure 2b). Each packaging bag was filled with precisely 60.00 ± 0.22 g of whole star anise arranged in a single-layer configuration. A fiber-optic temperature probe was inserted through a small aperture at the geometric center of each bag to enable temperature measurement within the star anise. Subsequently, bags were placed in the RF cavity and treated for 8 min or until reaching 70 °C (whichever occurred first) at 400 W input power and a 5 cm electrode gap. The initial and end sample temperatures before and after RF treatments were measured by the infrared camera within 30 s.

#### 2.5.3. Effect of Dual-Packaging Bag Placement on RF Heating Uniformity

The composite bag (one-sided plastic/one-sided aluminum foil) was selected as one of the target packaging materials for the dual-packaging experiments [25]. To investigate the effect of bag placement configurations on RF heating uniformity, 120 g of star anise was randomly divided into two parts with 60 g per bag. And the RF heating uniformity index was compared under the following conditions: (1) plastic-to-plastic face contact (both bags oriented with the plastic sides facing each other); (2) aluminum foil-to-aluminum foil back contact (both bags oriented with the aluminum foil sides facing away from each other); and (3) stacked configuration (bags placed directly superimposed with the plastic surface of the upper bag contacting the aluminum foil surface of the lower bag). Based on the preliminary experiment, a fiber-optic temperature sensor was inserted through an aperture at the geometric center of the bottom bag to measure the temperature of the star anise for each condition. Meanwhile, samples were heated for 8 min or until reaching 70 °C (whichever occurred first) at a fixed RF input power of 400 W with an electrode gap of 5 cm. For each condition, the surface temperatures before and after RF treatments of the top and bottom layers of star anise were measured by the infrared camera within 30 s.

### 2.6. RF Heating Uniformity Index

Since the rise in the mean and standard deviation of the sample temperature is entirely attributable to RF heating, by definition, the heating uniformity index (λ) contains the mean and standard deviation of the temperature (°C) distribution of the sample, as shown in the following expression [20]:

(1) $$\lambda =\frac{\sqrt{\sigma^{2}-\sigma_{0}^{2}}}{\mu -\mu_{0}}$$

where σ_0_ and σ are the standard deviation of surface temperatures in star anise before and after RF heating (°C), respectively. μ_0_ and μ are the average temperatures before and after heating (°C), respectively. The detailed definition process of λ values was described by Wang et al. (2005), and in this study λ was used as a relative indicator to compare the dispersion of surface temperature distributions among different RF heating treatments [26]. The λ has been widely used for evaluating experimental RF heating uniformity in multiple agriculture products and smaller λ values indicate the better RF heating uniformity [23,27,28].

### 2.7. Measurement of Dielectric Properties (DPs)

The measurement system for evaluating DPs (dielectric constant and loss factor) of star anise consisted of an RF impedance/material analyzer (E4991A, Agilent Technologies Inc, Palo Alto, CA, USA). Whole star anise, star anise seeds, and star anise pericarps were first ground separately and compressed into pellets (Φ = 7 mm, thickness 0.6 mm). The prepared samples were then loaded into the testing container, ensuring uniform distribution and the absence of visible air bubbles before measuring DPs.

The selected measurement range of frequencies was from 1 to 300 MHz with an interval of 13 MHz at 25 °C. The penetration depth (dp, m), defined as the distance from the material surface at which the incident power is attenuated to 1/e (e ≈ 2.718) of its initial value, can be expressed as follows [29]:

(2) $$d_{p}=\frac{c}{2\sqrt{2}\pi f{\left[\left|\varepsilon^{\prime }\right|\left(\sqrt{1+{\left(\frac{\left|\varepsilon^{\prime \prime }\right|}{\left|\varepsilon^{\prime }\right|}\right)}^{2}}-1\right)\right]}^{1/2}}$$

where *ε*′ and *ε*″ are the dielectric constant and loss factor of the sample (dimensionless), respectively, *f* is the frequency (Hz), and *c* is the speed of light in free space (3 × 10^8^ m/s).

### 2.8. Statistical Analysis

Mean values and standard deviations were obtained over the triplicate measurements. Significant difference was analyzed by Duncan’s multiple comparison tests with a probability value of *p* < 0.05 using statistical software (V21.0, SPSS Inc., Chicago, IL, USA).

## 3. Results and Discussion

### 3.1. Moisture Content and Dimensions of Star Anise

The moisture content for various parts of star anise is shown in Table 1. The moisture contents of de-seeded star anise and sample seeds were 5.70% ± 0.19% and 3.59% ± 0.11% (w.b.), respectively. Meanwhile, the moisture content of whole star anise (seeds retained) was 8.93% ± 0.09% (w.b.). Although seeds account for a small proportion, the overall moisture content was significantly lower. This agreed with the results reported by Guan et al., 2021 [30], in which the moisture content of the watermelon seed coat was significantly higher (*p* < 0.05) than that of the embryo. Similar trends were also observed in almonds and walnuts, where the moisture content of almond or walnut shells exceeded that of their respective kernels [31,32].

The dimensions of whole star anise (seeds retained) are listed in Table 2. The average longest diagonals, shortest diagonals and height of the star anise were 3.98, 3.37, and 0.92 cm, respectively. The critical height-to-diameter ratio of approximately 0.23 reflected the distinctly flattened morphology characteristic of star anise, with thickness being less than one-quarter of the maximum width. Based on electromagnetic field interaction theory, the flattened geometry of star anise caused electrode-to-product distances to vary significantly depending on the orientation of the eight-pointed protrusions relative to the electrodes. This spatial variation resulted in differential electric field exposure, creating uneven temperature distribution during RF heating.

### 3.2. Dielectric Properties of Star Anise

**Figure 3:**
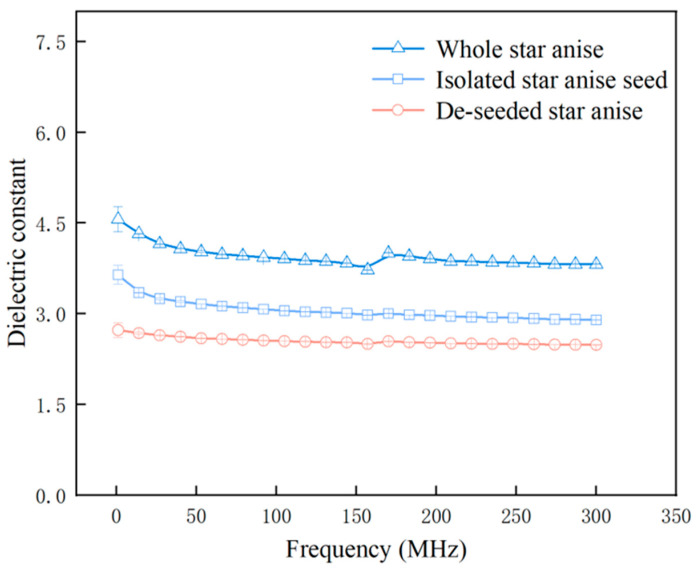
Frequency-dependent dielectric constant of different star anise components at 25 °C.

As shown in Figure 3, the dielectric constant for whole star anise, de-seeded star anise, and isolated star anise seed generally exhibited a decreasing trend with increasing frequency, eventually stabilizing in the high-frequency region. Similar results were observed in the DPs of peanut [9] and corn [33]. This behavior is consistent with previous studies on agricultural products, where dielectric properties are significantly influenced by frequency, moisture content, and tissue structure [34,35]. Across the tested frequency range, the magnitude of the dielectric constant followed the order: whole star anise > isolated star anise seed > de-seeded star anise. The highest dielectric constant of whole star anise can be attributed to its complex composite structure, including the pericarp, seeds, and internal air cavities, which facilitates stronger interfacial polarization. Isolated star anise seed, despite having a lower moisture content than de-seeded star anise (Table 1), exhibited a higher dielectric constant. This indicates that, for star anise, the effective dielectric response is not governed by moisture content alone, but is dominated by the combined effects of tissue microstructure and the density of polarizable components. Specifically, the isolated seeds possess a denser and more compact tissue microstructure with fewer air gaps and lower porosity, which increases the contribution of solid-phase polarization and interfacial polarization relative to the insulating effects of pores/air. In addition, the seeds contain higher relative concentrations of polar constituents (e.g., proteins and other polar biomolecules) [36], which can enhance the dielectric constant even when the absolute water content is lower. Conversely, de-seeded star anise, primarily composed of fibrous pericarp with higher porosity and weaker inherent polarity, showed the lowest dielectric constant [37]. Notably, a minor deviation from the general decreasing trend was observed for whole star anise around 150–170 MHz, which may be related to its internal tissue heterogeneity and associated interfacial polarization relaxation phenomena.

**Figure 4:**
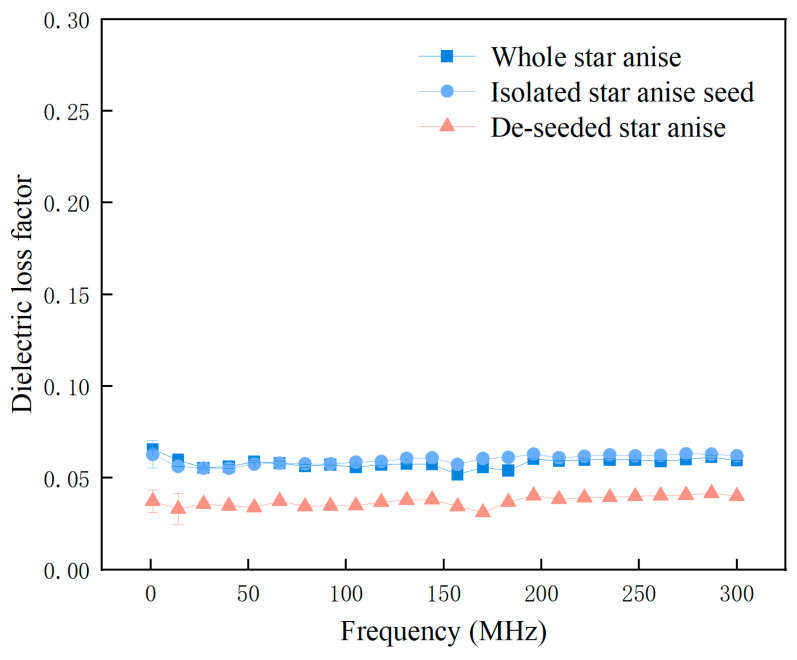
Frequency-dependent dielectric loss factor of different star anise components at 25 °C.

Figure 4 illustrates that the dielectric loss factor for all star anise remained relatively stable with minor fluctuations across the entire tested frequency range. Overall, the dielectric loss factor of isolated star anise seed and whole star anise was higher than that of de-seeded star anise, indicating their greater capacity for electromagnetic energy dissipation. This pattern mirrors the dielectric constant trend (Figure 3) and reinforces that the dense tissue microstructure and high concentration of polar constituents in seeds drive stronger electromagnetic coupling and energy conversion—a phenomenon not uniquely tied to moisture content. Indeed, existing studies on shelled peanuts have demonstrated that dielectric constant and dielectric loss factor both decrease with increasing moisture content [9], confirming that across different agricultural materials, tissue structure and chemical composition can dominate over moisture as primary determinants of dielectric loss. This aligns with the penetration depth presented in Table 3, where samples with higher dissipation factors (e.g., isolated star anise seed) generally showed smaller penetration depths, suggesting more concentrated energy absorption. The concentrated heating in high-loss materials like seeds may reflect two complementary mechanisms: (1) increased dielectric loss converts electromagnetic energy to heat more efficiently per unit depth, and (2) the dense tissue structure limits wave penetration, constraining energy deposition to a shallow zone. Conversely, de-seeded star anise, with its lower dielectric loss factor, exhibited larger penetration depths, indicating that the electromagnetic waves could propagate further into the material. As the frequency increased, the penetration depth also consistently decreased, indicating that the penetration ability of RF was reduced at higher frequencies. The results were consistent with the penetration depth of kiwifruit, tuna, and chestnut flour [38,39].

### 3.3. Determination of RF Input Power and Electrode Gap

**Figure 5:**
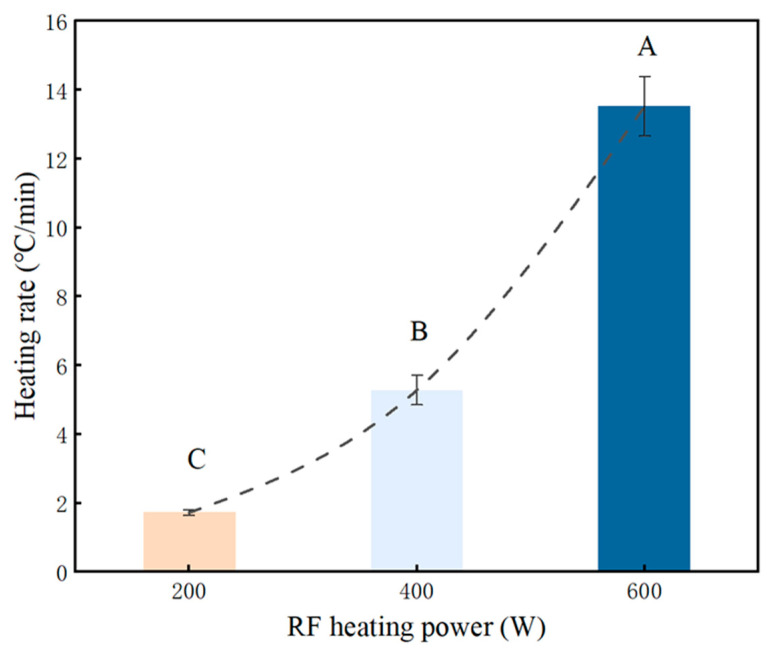
RF heating rate at different input powers. Note: The different capital letters indicate significant differences (*p* < 0.05) in heating rates at different RF input powers.

Figure 5 shows the heating rates of star anise at the geometric center of the PP container under the three selected input powers during RF processing. As the RF input power increased from 200 to 600 W, the electrical current with star anise in the container was low and decreased slightly from 1.72 to 13.53 °C/min. Similar results of heating rates in RF-treated cooked rice and minced chicken breast were also observed in Tian et al. [20,40], respectively. Since a heating rate of 3–5 °C/min is often used in the development of RF pasteurization processes for agricultural products, the input power of 400 W (5.28 °C/min) was chosen for further tests.

**Figure 6:**
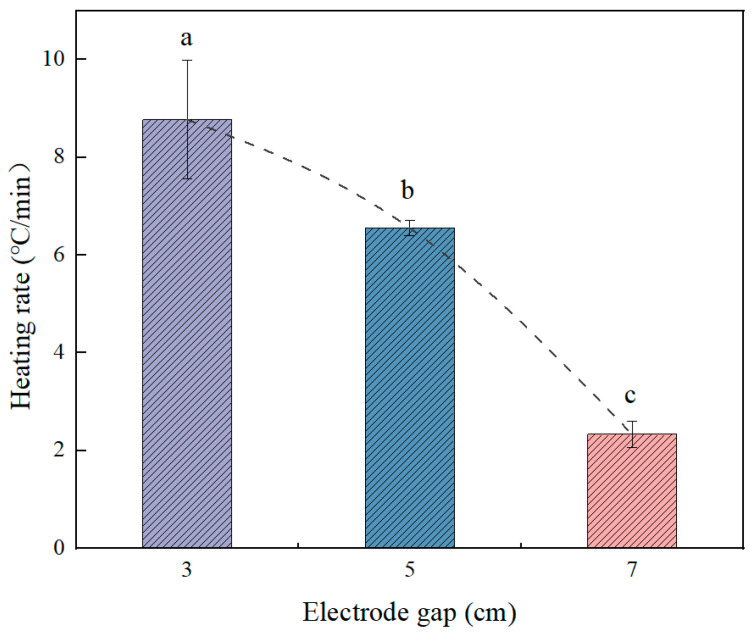
RF heating rate at different electrode gaps. Note: The different letters indicate significant differences (*p* < 0.05) in heating rates at different RF electrode gaps.

Figure 6 shows the heating rates of star anise at the geometric center of the PP container under the three selected electrode gaps during RF heating. The sample temperature increased almost linearly with heating time, while the heating rate decreased as the electrode gap increased. At the 7 cm electrode gap, the temperature increase after 8 min of RF heating was only about 40 °C, suggesting that this gap was insufficient to rapidly raise the sample to the target temperature. About 7.0 and 4.5 min were required for reaching the target temperature (70 °C) under the electrode gaps of 5 and 3 cm, respectively, resulting in heating rates of 6 and 9 °C/min. A similar trend was also reported in red pepper powder [41], liquid egg [42], and mung bean [43]. To ensure better quality of agricultural products and relatively short heating time, an electrode gap of 5 cm was selected for further tests to analyze the RF heating uniformity in the different arrangements and packaging types of star anise.

### 3.4. RF Heating Uniformity as Influenced by Star Anise Arrangements

Under the conditions of 400 W input power and a 5 cm electrode gap, the results for the effects of arrangements with sharp-edge contact, staggered sharp-edge arrangement, and reverse sharp-edge contact on RF heating uniformity are listed in Table 4.

The octagonal configuration with staggered sharp corners exhibits the smallest RF heating uniformity index (0.21 ± 0.012), demonstrating the best heating uniformity. Next is the sharp corner contact configuration (0.27 ± 0.027), while the reverse sharp corner contact configuration has the worst heating uniformity (0.30 ± 0.007). The better uniformity of the staggered arrangement is most likely associated with changes in the local electromagnetic and heat-transfer conditions within the container. The staggered arrangement minimizes void spaces within the container by offsetting the tips of adjacent star anise, thereby reducing the volume fraction occupied by air compared to star anise tissue. Since electromagnetic field intensity preferentially concentrates in low-permittivity regions, this reduction in air volume directly decreases overall electromagnetic field heterogeneity within the sample, promoting more uniform energy distribution. In contrast, the point-contact arrangement forms discrete contact regions at the sharp projections of the star anise, which can intensify localized electric field effects (commonly referred to as edge effects) and result in stronger temperature rise near those regions. Meanwhile, the staggered layout may provide more continuous inter-pod contact, offering better thermal conduction pathways that help smooth temperature gradients during the heating period. Notably, although the reverse point-contact arrangement produced the highest average temperatures, the corresponding temperature distribution was the least uniform [44], suggesting that specific orientations of the pods could promote preferential energy accumulation and localized heating. Meanwhile, in the future, computer simulations can be employed to further investigate the differences in RF heating behavior and electromagnetic fields attributed to the orientation of star anise. Therefore, the staggered arrangement was selected for subsequent packaging optimization experiments because it provided better heating uniformity under the tested RF conditions. It should be noted that the RF heating uniformity index alone cannot fully confirm microbiological safety because minimum-temperature mapping and cold-spot tracking were not performed. Further cold-spot validation and microbial challenge or surrogate tests are needed to ensure target lethality while minimizing overheating.

### 3.5. RF Heating Uniformity as Influenced by Packaging Materials

The RF heating rate and heating uniformity index of star anise in kraft paper packaging bags, double-sided plastic packaging bags, and packaging bags with one side plastic and the other side of 0.16 mm aluminum foil are listed in Table 5. The packaging bag with plastic and aluminum foil exhibited the fastest heating rate (7.135 ± 0.35), while kraft paper bags and plastic bags showed the slowest heating rates. The effect of the plastic/aluminum foil laminated packaging on RF heating behavior is mainly attributed to the high electrical conductivity of aluminum foil and the resulting electromagnetic boundary effects. As a conductive material, aluminum foil can modify the electromagnetic boundary conditions at the metal–dielectric interface. In contrast, the lower final temperature observed in the aluminum foil laminated package (61.19 ± 1.86 °C) may be related to the reflection and shielding effects of the aluminum layer, which can alter RF electric field distribution and reduce effective energy penetration into some regions of the product.

The packaging material effects on RF heating behavior reflect complex interactions between the dielectric characteristics of star anise and the electromagnetic boundary conditions introduced by different packaging materials [45]. The composite plastic/aluminum foil structure, despite not producing the highest final temperatures, achieved better RF heating uniformity (lowest SD) and the fastest heating rates. There are two reasons for the reduction in temperature differences. One reason was that the thin aluminum layer partially reflects electromagnetic waves, concentrating RF energy within the product layer rather than allowing it to disperse diffusely into the surrounding air [45]. Another reason was that the heterogeneous plastic–metal interface created controlled electromagnetic boundary effects that can enhance rather than inhibit uniform field distribution at intermediate thicknesses according to electromagnetic field theory [27]. The dominant role of aluminum’s electrical conductivity (not thermal conductivity) was confirmed by the observed experimental patterns: the fastest heating rate and lowest temperature variance (SD) demonstrate that electromagnetic field shaping, not thermal diffusion, drives the improved heating behavior. The effect of the aluminum foil laminated package on RF heating behavior should be mainly attributed to its high electrical conductivity rather than its thermal conductivity. As a conductive layer, aluminum foil can act as a field-shaping boundary, leading to shielding, reflection, and redistribution of the RF electric field near the metal–dielectric interface [23]. This may alter the local electric field intensity and RF energy absorption in the adjacent spice samples, thereby affecting the temperature distribution. In contrast, kraft paper and plastic bags are dielectric packaging materials and do not provide the same conductive-boundary effect as aluminum foil. Based on the more uniform temperature distribution and higher heating rate observed experimentally, the plastic/aluminum foil laminated bag with a thickness of 0.16 mm was selected for subsequent experiments.

### 3.6. RF Heating Uniformity as Influenced by Packaging Bag Placement

Data presented in Table 6 show the average temperature, standard deviation of temperature distribution, RF heating rate, and uniformity index in star anise under different placements of the two packaging bags. For the stacked configuration, the temperature differential between top and bottom layers was approximately 16.5 °C. In contrast, both the face-to-face and back-to-back configurations achieved substantially higher temperatures. Notably, the face-to-face and back-to-back configurations showed surface temperature differences of approximately 11.8 °C and 31.4 °C, respectively. The only exception was the stacked configuration, in which the bottom layer was warmer. This temperature inversion pattern can be attributed to the electromagnetic properties of the interfacing materials. In the stacked configuration, the plastic surface of the upper bag contacts the aluminum foil surface of the lower bag, creating a heterogeneous dielectric interface. This heterogeneous interface may preferentially concentrate electromagnetic energy in the lower-layer region, resulting in elevated lower-layer temperatures but relatively poor upper-layer heating. Conversely, in the face-to-face and back-to-back configurations where identical material surfaces contact each other (plastic-to-plastic or foil-to-foil), the more symmetric electromagnetic field distribution permits relatively uniform energy delivery to both layers.

The face-to-face configuration demonstrated statistically better heating uniformity at the top and bottom layers (Table 6) compared to other configurations (*p* < 0.05). This elevated variability in stacked configuration suggests significant spatial temperature gradients, indicating that certain regions within the lower layer achieved substantially higher temperatures than others, creating unacceptable microbiological safety risks if cold spots remain below the pasteurization target. The back-to-back configuration, while producing high absolute temperatures (top layer: 103.27 °C), exhibited uniformity indices comparable to or poorer than the face-to-face configuration at the bottom layer (λ = 0.24 ± 0.044) and substantially elevated temperature extremes, suggesting potential quality degradation from excessive localized heating.

These uniformity results demonstrated that placement configuration exerts profound effects on temperature distribution homogeneity. The superior uniformity of the face-to-face configuration (λ = 0.10 top layer) likely reflects symmetric electromagnetic field distribution created by the plastic-to-plastic interface. When identical dielectric surfaces contact each other, the electromagnetic field penetrates symmetrically through both layers, avoiding the asymmetric field concentration that occurs at heterogeneous plastic-to-metal interfaces (as in the stacked configuration). This symmetry minimizes localized electromagnetic energy accumulation and associated temperature hotspots, promoting more homogeneous heating throughout both layers. Moreover, the back-to-back and stacked configurations achieve localized temperatures exceeding 100 °C in some regions, creating a risk of thermal damage to thermolabile bioactive compounds in star anise (e.g., anethole, antioxidants, antimicrobial essential oils). The face-to-face configuration’s more uniform heating minimizes such extreme temperature zones. Thus, although the stacked configuration might achieve faster lower-layer heating under specific conditions, the face-to-face configuration’s statistically better heating uniformity and superior quality preservation support safer processing conditions, making it a promising choice for commercial RF pasteurization of packaged star anise.

## 4. Conclusions

The moisture content of star anise varied significantly among morphological fractions. Whole star anise showed the highest moisture content, followed by de-seeded star anise, while star anise seeds exhibited the lowest moisture content. RF heating performance depended strongly on processing parameters. Increasing RF input power and decreasing electrode spacing accelerated the heating rate. With the optimized operating conditions of 400 W and an electrode gap of 5 cm, heating uniformity was further improved by product and package configuration. Among the tested arrangement patterns, the staggered arrangement provided the better uniformity with the lower λ. Using a plastic/0.16 mm aluminum foil composite bag resulted in faster heating and improved uniformity compared with kraft paper or plastic bags alone. For dual-bag placement, the face-to-face configuration achieved the most favorable heating homogeneity, whereas the stacked configuration led to the largest temperature differential between the top and bottom layers. However, because the commercial value of star anise is strongly associated with its volatile essential oils and characteristic aroma, the optimized 8 min RF treatment should be further validated by GC-MS analysis of volatile compounds and sensory evaluation before commercial or industrial application. Future work should also include microbiological challenge tests and pilot-scale validation under more realistic processing conditions.

## Figures and Tables

**Table 1:** Moisture content of seedless anise, intact anise, and anise seeds.

	Schematic Diagram	Moisture Content (%, w.b.)
De-seeded star anise	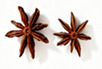	5.70 ± 0.19 B ^#^
Isolated star anise seed	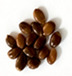	3.59 ± 0.11 A
Whole star anise	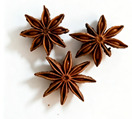	8.93 ± 0.09 C

# Different letters within the same row indicate significant differences between corresponding columns (*p* < 0.05).

**Table 2:** Mean values of longest diagonal, shortest diagonal, and height of typical star anises, and corresponding schematic diagrams.

	Schematic Diagram	Value (cm)
The longest diagonal	** 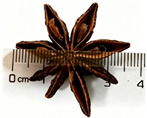 **	3.98 ± 0.36 A *
The shortest diagonal	** 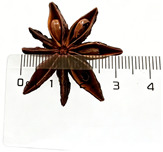 **	3.37 ± 0.30 B
Thickness	** 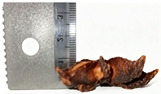 **	0.92 ± 0.09 C

* Different letters within the same row indicate significant differences between corresponding columns (*p* < 0.05).

**Table 3:** Dielectric constant, loss factor, and penetration depth of different sections of an octagon at frequencies of 13, 27, and 40 MHz.

Sample	Frequency (MHz)	Dielectric Constant	Dielectric Loss Factor	Penetration Depth (m)
Whole star anise	13	4.32 ± 0.0271 A	0.0517 ± 0.0031 B	148.05 ± 8.7751 A
	27	4.16 ± 0.0057 B	0.055 ± 0.0012 A	64.73 ± 0.53 B
	40	4.08 ± 0.0047 C	0.0562 ± 0.001 A	42.91 ± 0.792 C
Isolated star anise seed	13	3.4 ± 0.0022 A	0.0569 ± 0.0022 A	119.06 ± 4.5679 A
	27	3.26 ± 0.0051 B	0.0553 ± 0.0025 A	57.635 ± 0.1344 B
De-seeded star anise	40	3.2 ± 0.0075 C	0.0552 ± 0.0005 A	38.62 ± 0.3818 C
	13	2.73 ± 0.0003 A	0.0394 ± 0.0012 A	154.115 ± 4.6457 A
	27	2.65 ± 0.0013 B	0.0358 ± 0.0016 B	80.43 ± 3.4931 B
	40	2.62 ± 0.0002 C	0.035 ± 0.0005 B	55.185 ± 0.7849 C

Different letters within the same row indicate significant differences between corresponding columns (*p* < 0.05).

**Table 4:** Average final test temperatures (*T*_ave_), standard deviation (SD) of temperature distributions, and RF heating uniformity index (*λ*) for three permutations.

	Sharp Corner Contact	Sharp-Angled Derangement	Reverse Cusp Contact
*T_ave_* (°C)	59.70 ± 0.25 B ^#^	74.49 ± 1.26 A	77.35 ± 0.90 A
SD (°C)	9.49 ± 0.91 B	11.00 ± 0.36 B	15.74 ± 0.66 A
*λ*	0.27 ± 0.027 AB	0.21 ± 0.012 B	0.30 ± 0.007 A
Thermal imaging of top surface	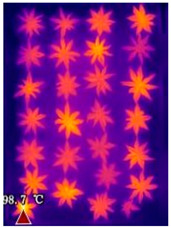	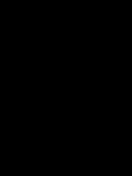	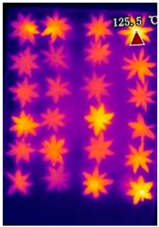

# Different letters within the same row indicate significant differences between corresponding columns (*p* < 0.05).

**Table 5:** The average temperature (*T*_ave_), standard deviation (SD) of surface temperature distribution, and RF heating uniformity index (*λ*) for the three materials.

	Kraft Paper	Plastic	0.16 mm Aluminum Foil
*T_ave_* (°C)	75.03 ± 1.36 A ^#^	71.55 ± 0.77 A	61.19 ± 1.86 B
SD (°C)	8.49 ± 1.42 A	8.55 ± 1.10 A	6.54 ± 1.25 A
*λ*	0.17 ± 0.032 A	0.18 ± 0.021 A	0.18 ± 0.025 A
Heating rate (°C/min)	5.245 ± 0.59 B	4.605 ± 0.18 B	7.135 ± 0.35 A

# Different letters within the same row indicate significant differences between corresponding columns (*p* < 0.05).

**Table 6:** The average temperature (*T*_ave_), standard deviation (SD) of surface temperature distribution, and RF heating uniformity index (*λ*) for the stacked, face-to-face, and back-to-back formats.

	Layer	Stacked	Face-to-Face	Back-to-Back
*T_ave_* (°C)	Top Bottom	43.97 ± 0.59 B ^#^ 60.47 ± 4.31 C	99.79 ± 0.91 A 87.97 ± 1.67 A	103.27 ± 2.30 A 71.88 ± 2.04 B
SD (°C)	Top Bottom	3.52 ± 0.43 A 11.51±0.90 A	7.82 ± 0.88 A 10.70 ± 0.77 A	14.622 ± 6.05 A 11.45 ± 2.55 A
*λ*	Top Bottom	0.18 ± 0.017 A 0.32 ± 0.036 A	0.10 ± 0.011 A 0.17 ± 0.008 B	0.15 ± 0.071 A 0.24 ± 0.044 AB
Heating rate (°C/min)	Bottom	16.15 ± 0.83 A	4.31 ± 0.17 B	5.48 ± 0.08 B

# Different letters within the same row indicate significant differences between corresponding columns (*p* < 0.05).

## Data Availability

The raw data supporting the conclusions of this article will be made available by the authors on request.
